# Midkine level may be used as a noninvasive biomarker in Crohn’s disease

**DOI:** 10.3906/sag-1904-167

**Published:** 2020-04-09

**Authors:** Murat KEKİLLİ, Alpaslan TANOĞLU, Fatih KARAAHMET, Zeynal DOĞAN, Murat CAN, Abdurrahim SAYILIR, Başak ÇAKAL, Tolga DÜZENLİ, Yavuz BEYAZIT

**Affiliations:** 1 Department of Gastroenterology, Health Sciences University Ankara Training and Research Hospital, Ankara Turkey2; 2 Department of Gastroenterology, Health Sciences University Sultan Abdulhamid Han Training and Research Hospital, İstanbul Turkey3; 3 Department of Biochemistry, Faculty of Medicine, Zonguldak Karaelmas University, Zonguldak Turkey4; 4 Department of Gastroenterology, Medical Park Karadeniz Hospital, Trabzon Turkey5; 5 Department of Gastroenterology, Çanakkale Onsekiz Mart University, Çanakkale Turkey

**Keywords:** Midkine, inflammatory bowel disease, Crohn’s disease, biomarker, inflammation

## Abstract

**Background/aim:**

Crohn’s disease (CD) is a kind of inflammatory bowel disease. Midkine (MDK) is an endogenous inflammatory marker. We aimed to investigate the relationship between MDK levels and inflammation and hence determine whether MDK can be used as a noninvasive biomarker in active CD.

**Materials and methods:**

Sixty-five consecutive patients over the age of 18 with CD and 36 healthy controls were included in this study. CD patients’ venous blood samples were taken before treatment. Serum MDK levels were determined in human plasma samples by enzyme-linked immunosorbent assay (ELISA) method.

**Results:**

The mean age of the study patients was 44.8 ± 12.5 years, 35 patients were female, and 30 were male. Of these 65 patients, 37 had active CD and 28 were in the remission phase. MDK levels were significantly higher in active and remission CD than in healthy controls (P = 0.01, P = 0.038, respectively).

**Conclusion:**

We report that there is an association between MDK levels and CD activation, and therefore with enhanced inflammation. MDK levels were significantly correlated with inflammatory indices. In line with our findings, we suggest the theory that MDK inhibitors may be useful in treating Crohn’s disease.

## 1. Introduction

Crohn’s disease (CD) is a kind of inflammatory bowel disease (IBD) that can lead to various comorbidities [1]. Besides intestinal system involvement, CD may lead to a variety of complications outside of the gastrointestinal system (GIS) (e.g., anemia, skin disease, arthritis, inflammation of the eyes, bone disease, kidney stones) [1–3]. Immunity has a pivotal role in the progress of CD, and understanding the immunopathogenesis of CD in a detailed manner is crucial for management of the complications and of course for developing efficient treatment regimens. Although it has been proposed that Crohn’s disease is a primary T-cell autoimmune disorder [4,5], the exact underlying pathogenesis is still unclear.

Midkine (MDK), also called neurite growth-promoting factor 2 (NEGF2), is a basic heparin-binding growth factor of low molecular weight encoded by the *MDK* gene. Although MDK expression is limited mainly to certain tissues, it is strongly stimulated in inflammation, oncogenesis, and tissue repair [6]. MDK induces IL-12 production, thus aggravating the inflammatory cascade, which can subsequently lead to important actions such as cell proliferation, cell migration, and angiogenesis via innate immune mechanisms [6,7]. Furthermore, it has been shown that the IL-12- and IL-23-driven Th1 signaling pathway is inevitably involved in the inflammatory course of CD [8,9]. Moreover, high local expression of TNF-α and IL-1β in active IBD also induces MDK expression [10].

Altogether, it can be suggested that MDK is an endogenous inflammatory marker involved in the GIS that is related to TNF-α, IL-1β, IL-12, and IL-23 production. In this research we aimed to determine whether MDK could be used as a noninvasive biomarker in active CD.

## 2. Materials and methods

This prospective study was carried out at the Ankara Education and Research Hospital, Department of Gastroenterology, after obtaining the permission of the local ethics committee. Sixty-five consecutive newly diagnosed CD patients over the age of 18, who were admitted to our hospital between September 2015 and October 2017, and 36 age- and sex-matched healthy controls were included in this study. Diagnosis and treatment were based on the European Crohn´s and Colitis Organization (ECCO) guidelines [11].

In this study, the exclusion criteria included having extraintestinal manifestations of Crohn disease, coinfection of viral hepatitis, HIV infection, other autoimmune liver diseases, metabolic disease, cardiac disease, renal disease, diabetes mellitus, atherosclerotic disease, chronic infection, history of hypertension, malignancy, autoimmune disorders, rheumatic disease, hematological disease, chronic obstructive lung disease, and solid organ transplantation, as well as patients taking drugs such as aspirin, NSAIDs, warfarin, heparin, steroids, antidiabetics, hyperlipidemics, and antihypertensives. All participants gave written informed consent for this IBD study.

All CD patients’ venous blood samples were obtained before patients were given any kind of therapeutic agent. Using venous blood samples, determinations were made of white blood cell (WBC) count, hemoglobin, platelet (PLT) count, C-reactive protein (CRP), erythrocyte sedimentation rate (ESR), albumin, creatinine, urea, alanine aminotransferase (ALT), aspartate aminotransferase (AST), and MDK levels. Demographic, clinical, and laboratory data were collected for all subjects and the Crohn’s Disease Activity Index (CDAI) was calculated for clinical disease activity.

Venous blood samples for biochemical parameters were obtained by venipuncture. Moreover, serum MDK levels were tested by enzyme-linked immunosorbent assay (ELISA) method using a commercial ELISA kit (Boster Biological Technology, Pleasanton, CA, USA), according to the manufacturer’s instructions. This particular immunoassay utilizes the quantitative technique of sandwich ELISA. MDK tests were accomplished with an Elx800 automatic ELISA reader (BioTek, Winooski, VT, USA) and results were calculated from the calibration curve. Sensitivity of the MDK assay was <10 pg/mL. The coefficients of intraassay and interassay variation of MDK were 4.6%–7.3%.

Statistical tests were carried out using SPSS 18 (SPSS Inc., Chicago, IL, USA). First, normality was determined by the Kolmogorov–Smirnov test. Values are presented as mean ± standard deviations or as median and range due to normality tests. Unpaired Student t-tests or Mann–Whitney U tests were used again owing to the Kolmogorov–Smirnov test. For more than two groups, comparison of numerical variables was initially performed using one-way ANOVA or the Kruskal–Wallis test.

Receiver operating characteristic (ROC) curve analysis was used to identify optimal cut-off values of MDK, WBC, CRP, ESR, and PLT. Moreover, ROC analysis was used to determine maximum sensitivity and specificity for differentiation of active Crohn’s from inactive Crohn’s. P < 0.05 was accepted as statistically significant.

## 3. Results

The mean age of the patients in this current research was 44.8 ± 12.5 years, and 30 patients were male. Of the 65 patients, 37 had active CD. When the patients were divided into two groups based on active disease and remission, no statistically significant difference between the two groups for baseline age, sex, hemoglobin, albumin, creatinine, urea, ALT, or AST was observed. Also, there was no statistical difference in disease duration between the two groups. Mean baseline CDAI was significantly elevated in the active CD group compared to the remission CD group (P < 0.001). The demographic characteristics, laboratory parameters, and activity index and behavior of the CD patients in the study population are summarized in Table 1.

**Table 1:** Demographic features and laboratory values of the Crohn patients and controls.

	Control group(n: 36)	Activation ofCrohn’s (n: 37)	Remission ofCrohn’s (n: 28)	P-value
Age (years)	42.8 ± 11.53	42.4±13.1	47.1 ± 12.7	0.274
SexFemaleMale	26 (72.2%) 10 (27.8%)	24 (64.9%) 13 (35.1%)	11 (39.3%)17 (60.7%)	0.022
Hemoglobin (g/dL)	13.7 ± 1.3	12.9 ± 1.8	13.1 ± 1.7	0.120
Urea (mg/dL)	28.5 (10–50)	28 (10–52)	26 (8–79)	0.892
Creatinine (mg/dL)	0.8 (0.6–1.2)	0.9 (0.5–1.3)	0.9 (0.5–1.8)	0.051
ALT (IU/mL)	19 (8–40)	19 (12–66)	16 (10–56)	0.487
AST (IU/mL)	20 (10–59)	22 (10–46)	21 (15–34)	0.926
Albumin (mg/dL)	4.4 ± 0.2	4.3 ± 0.4	4.4 ± 0.3	0.679
Disease duration (years)	-	2 (0.4–11)	2.6 (0.6–4)	0.928
CDAI	-	177 (153–462)	49.5 (9–148)	<0.001
Localization of diseaseIlealColonicIleo-colonic		4 (10.8%)14 (37.8%)19 (51.4%)	10 (35.7%)8 (28.6%)10 (35.7%)	

Data are presented as median (range) or mean ± SD.

Mean baseline WBC, CRP, and MDK levels were significantly higher in the active CD group compared to the control group (P < 0.001). There was no statistical significance in PLT count and ESR among these two groups. Comparisons of serum MDK levels and other inflammation markers between Crohn’s patients and controls are summarized in Table 2.

**Table 2:** Comparison of serum midkine levels and other inflammation markers between Crohn’s patients and controls.

	Control group(n: 36)	Activation ofCrohn’s (n: 37)	Remission ofCrohn’s (n: 28)	P
WBC (/mm3 × 103)	6.5 ± 1.1	9 ± 3.6	7.7 ± 2.1	0.001*
Platelet (/mm3 × 103)	249 ± 62	272 ± 84	284 ± 74	0.172
CRP (mg/L)	0.3 (0.3–6.5)	1.24 (0.1–12)	0.3 (0.1–4)	<0.001**
ESR (mm/h)	8 (2–36)	21 (2–56)	13 (2–45)	0.065
Midkine	154.8 ± 76.1	281 ± 109.7	270.8 ± 90.8	<0.001***

Data are presented as median (range) or mean ± SD. *Active vs. remission, P = 0.049; active vs. control, P < 0.001; remission vs. control, P = NS. **Active vs. remission and control, P < 0.001; remission vs. control, P = 0.502. ***Control vs. active and remission, P < 0.001, active vs. remission, P = 0.666.

**Figure 1:**
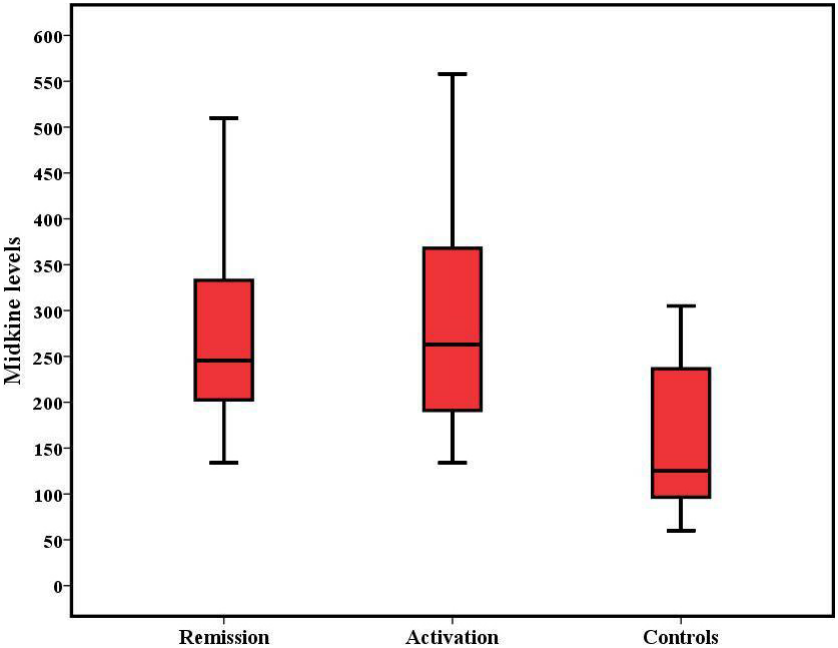
MDK levels in Crohn’s disease and control patients.

When we compared serum inflammatory marker levels (ESR, MDK, and PLT) between the control group and active CD and remission groups, no statistically signiﬁcant correlation was observed (P = 0.065, P = 0.666, P = 0.172, respectively). MDK levels were significantly higher in active and remission CD patients than controls (P = 0.01, P = 0.038, respectively) (Figure 1).

**Figure 2:**
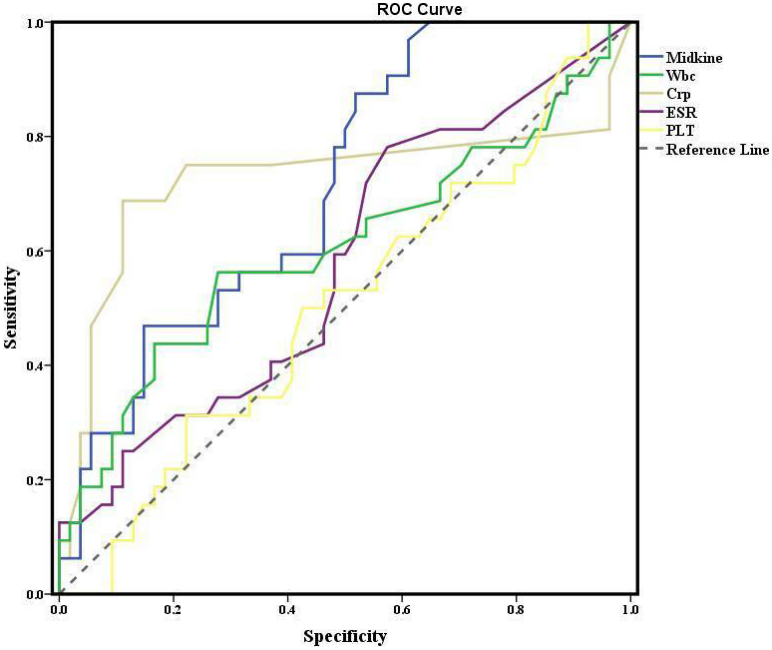
The overall accuracy and ROC analysis.

The sensitivity of MDK levels in active CD was 54.1% with specificity of 53.6%. Also, the positive predictive value of MDK level was 60.6% in active CD. Sensitivity and specificity of serum MDK levels and other inflammation markers are shown in Table 3 for differentiating active CD from remission. Additionally, the overall accuracy and ROC analysis are shown in Figure 2.

**Table 3:** Overall accuracy and ROC analyses of midkine and other inflammation markers to differentiate activation of Crohn’s from remission.

	AUC	Sensitivity (%)	Specificity (%)	NPV (%)	PPV (%)	Overall accuracy
Midkine (cutoff: 255.5)	0.525	54.1	53.6	46.9	60.6	53.8
WBC (cutoff: 7850)	0.601	62.2	60.7	54.8	67.6	61.5
CRP (cutoff: 0.55)	0.756	77.8	83.2	74.2	84.8	78.4
ESR (cutoff: 11)	0.497	57.6	41.7	41.7	57.6	50.8
PLT (cutoff: 263500)	0.466	56.8	46.4	44.8	58.3	52.5

CRP: C-reactive protein, ESR: erythrocyte sedimentation rate, WBC: white blood cells, AUC: area under the curve, PPV: positive predictive value, NPV: negative predictive value.

## 4. Discussion

In this study we report that there is an association between MDK levels and CD itself, and therefore with enhanced inflammation. There are many cytokines that can be associated with gut inflammation. It has been shown that among patients with CD, higher TNF-α, IL-1β, and IL-6 levels were related to disease activation and inflammation [7,12]. Some other studies reported that IL-12, IL-13, IL-17, and IL-23 have a central role in CD [1,8,13,14]. TNF-α, IL-1β, IL-12, and IL-13 are overexpressed through an immune reaction that is predominated by type 1 helper T (Th1) cells. These cytokines are associated with elevated MDK concentrations. Thus, it can definitely be concluded that MDK is an endogenous inflammatory agent involved in gut activation mediated by TNF-α, IL-1β, IL-12, IL-13, IL-17, and IL-23. In line with these suggestions, a higher MDK level in a CD patient can be attributed to higher rates of clinical activation and enhanced inflammation. Also, in our current study, MDK levels were significantly correlated with inflammatory indices including CRP and WBC, which is in line with the literature [15].

Immunity has been traditionally considered to play a pivotal role in CD activity and behavior. In particular, due to immune responses, CD-specific damage is localized to the gut. However, although being studied globally, characteristic mechanisms of CD extraintestinal manifestations are largely unknown. In light of these facts, we could not obtain sufficient evidence to draw firm conclusions along with CD disease activation, disease localization, disease behavior, and MDK levels in our current study. Moreover, whether higher MDK levels, associated with a fistula or any other complication, could be a predictive biomarker of surgery continues to remain uncertain.

MDK is primarily studied in conditions with malignancy, inflammation, and preexisting peripheral vascular disease or ischemia [6,7]. MDK was studied in patients with ulcerative colitis (UC) by Krzystek-Korpacka et al. in 2009 [16]. They studied MDK in 93 UC patients and 108 healthy subjects. The authors found that MDK was significantly higher (P < 0.0001) in inactive and active UC compared to controls. Finally, they concluded that UC was clearly associated with increased circulating MDK, which corresponds with clinical, endoscopic, inflammatory, and angiogenic activity, and with anemia [16].

Krzystek-Korpacka et al. first studied the relationship between MDK levels and CD [15]. They investigated MDK levels and other inflammatory indices in 91 CD patients and found that MDK could be used as a biochemical marker of active CD with sensitivity and specificity of 86% and 97%, respectively. In the same study, CRP had sensitivity and specificity of 83% and 92%, respectively. To the best of our knowledge, our paper is the second one that has investigated the relationship between MDK levels and active CD. The most important differences of this current paper from the previous study were our rigid patient exclusion criteria and that our active CD patients had mild or moderate activation rather than severe activation.

In our study, the sensitivity of MDK level was 54.1% among active CD patients with a specificity of 53.6%. Also, there was no statistical significance of MDK level between the active CD group compared to remission. This finding may be attributed to the relatively small sample size of the study. Moreover, in this current study, most active CD patients had mild or moderate activation. Although MDK was increased among all CD patients, we cannot say that MDK can be used as a highly sensitive marker of active CD. Finally, our rigid patient exclusion criteria may have caused this differentiation between these two studies.

Current studies have reported that MDK has a role in cancer disease in several organs. In a study conducted on breast cancer and MDK levels, it was concluded that MDK levels may be more beneficial than currently used tumor markers in the diagnosing and discriminating of primary and metastatic breast cancer [17]. We may also speculate that MDK might enter into the blood circulation from tumor tissues, and that plasma MDK levels could be clinically relevant in patients with IBD-associated colonic malignancy.

A few limitations exist in our study. First, the study population mostly consisted of nonstricturing and nonpenetrating ileo-colonic patients, which limited the variety of the patients. This limitation may not have severely affected the results, but it reveals a lack of homogeneity. Secondly, only MDK, ESR, and CRP were studied in view of inflammation in this research. Thirdly, no comparison with medical treatment was performed. Additionally, this was a single-center study and also the relatively small sample size may have posed limitations in this study.

Finally, there was a strong association of MDK level and severity of intestinal inflammation among CD patients. Studying the MDK levels together with other specific inflammatory markers (e.g., IL-13, IL-12) would emphasize the advantages of MDK (e.g., efficacy, specificity) in the prediction of gut inflammation, especially in CD but also in UC patients. In line with our findings, we suggest the theory that MDK inhibitors may be useful in treating Crohn’s disease.

## Ethics committee approval

Ethics committee approval was received for this study from the institutional ethics committee.

## Financial disclosure

The authors declared that this study received no financial support.
